# Retraction: Long non-coding RNA ELFN1-AS1 promoted colon cancer cell growth and migration via the miR-191-5p/special AT-rich sequence-binding protein 1 axis

**DOI:** 10.3389/fonc.2023.1322464

**Published:** 2023-11-27

**Authors:** Frontiers Editorial Office

Please find the retraction statement below:

Following publication, concerns were raised regarding the integrity of the images in the published figures.

Following the provision of raw data by the authors, the Chief Editor concluded that the article’s conclusions and assertions were not sufficiently supported by the findings from the material provided; therefore, the article has been retracted.

This retraction was approved by the Chief Editor of Frontiers in Oncology and the Chief Executive Editor of Frontiers.

